# The added value of streamline visualization in the evaluation of left atrioventricular valve flow and left ventricular diastolic function with 4DFlow MRI

**DOI:** 10.1186/1532-429X-16-S1-P353

**Published:** 2014-01-16

**Authors:** Emmeline Calkoen, Arno Roest, Lucia J Kroft, Pieter J van den Boogaard, Monique R Jongbloed, Rob J van der Geest, Albert de Roos, Jos J Westenberg

**Affiliations:** 1 Pediatric cardiology, LUMC, Leiden, Netherlands; 2 Radiology, LUMC, Leiden, Netherlands; 3 Cardiology, LUMC, Leiden, Netherlands

## Background

4DFlow MRI with retrospective valve tracking allows trans-valvular blood flow quantification. Valve tracking usually follows the anatomical annulus but does not take into account the inflow direction through the valve, which might lead to substantial errors when opening of the valve leaflets is restricted after surgery. We aimed to evaluate the added value of streamline visualization in the characterization and quantification of trans-left atrioventricular valve (LAVV) blood flow and assessment of left ventricular (LV) diastolic function in 4DFlow MRI.

## Methods

**Figure 1:**
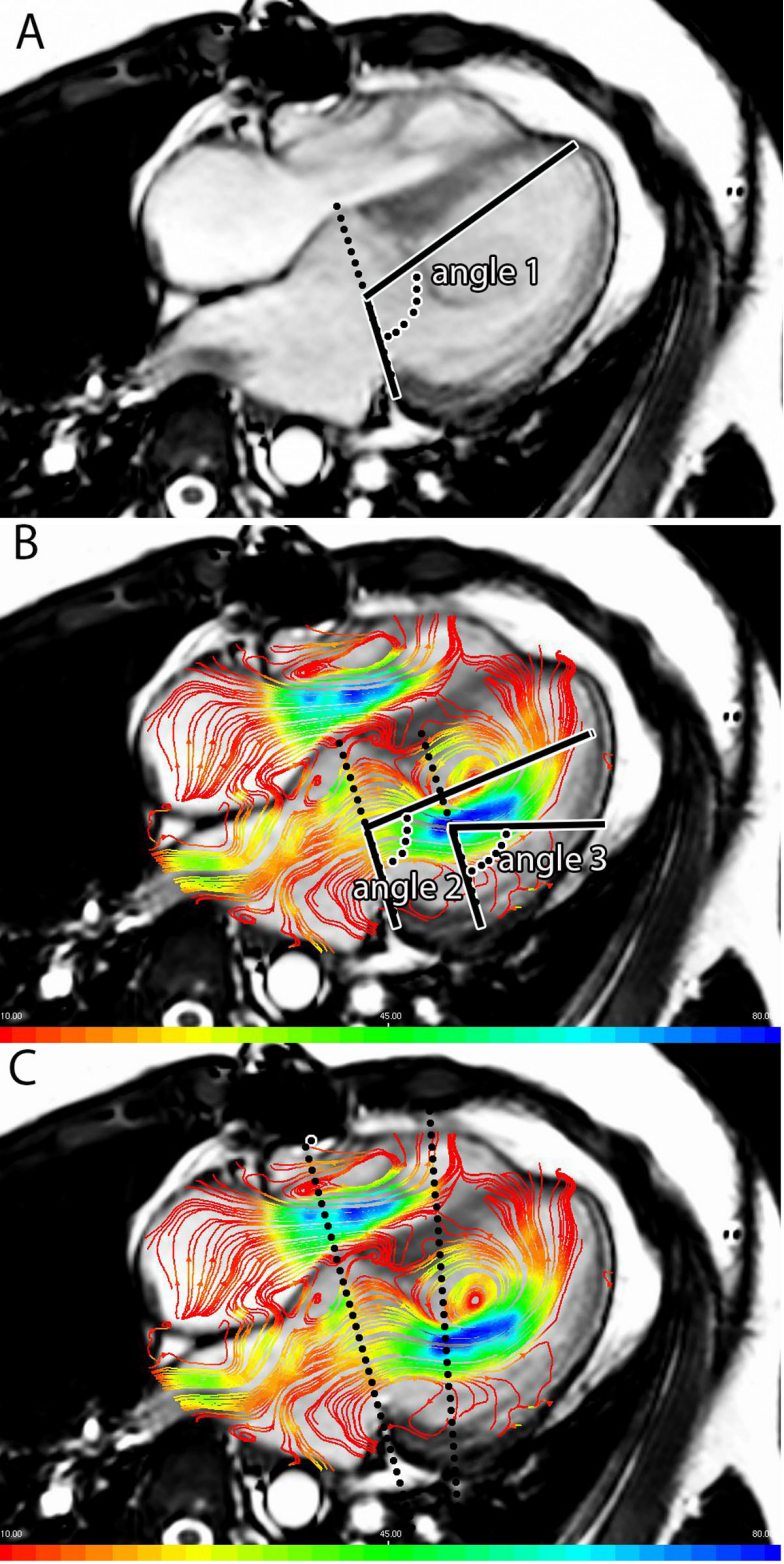
**Illustrates the angle between annulus and long axis (angle 1) and the angles between annulus and inflow at annulus level (angle 2) and peak velocity level (angle 3)**. In panel C the positioning of the two reformat planes at annulus level and at peak velocity level angulated perpendicular to the inflow are shown.

In 25 patients with a history of corrected atrioventricular septal defect (AVSD) (mean age 23 ± 10 years) and 25 healthy subjects (21 ± 11 years), whole-heart 4DFlow MRI was performed at 3T (Ingenia, Philips, The Netherlands), during free breathing, with three-directional velocity encoding of 150 cm/s, spatial resolution 2.3 × 2.3 × 3.0-4.2 mm3, flip angle 10°, echo-time 3.2 ms and repetition-time 7.7 ms. In a 2- and 4-chamber view, streamline visualization was used to determine the inflow direction. The angle between LV long-axis (i.e., line through the center of the annulus and apex) and annulus was measured in the 4-chamber view (Figure 1A). Secondly, the inflow direction at early (E) and late (A) peak filling was measured at annular level and at the level of peak velocity distal to the annulus (Figure 1B). Trans-LAVV flow volume and velocity were assessed using velocity mapping from two reformat planes; one aligned with the annulus and the other angulated perpendicular to the inflow direction (Figure 1C).

## Results

Results from angle measurements and trans-LAVV velocity mapping are presented in Table 1. In patients the inflow at peak velocity level was significantly more laterally directed than at annulus level (19° versus 10°, p = 0.003). At either sampling position, LAVV effective forward flow was not statistically significantly different from aortic flow. However in patients, better agreement (stronger correlation and smaller coefficient of variation) was found between LAVV-flow at peak velocity level and aortic flow than at annular level. Furthermore, velocity mapping at the level of peak velocity with reformat plane oriented perpendicular to the inflow direction resulted in statistically significant changes in inflow parameters (Table 1) in healthy subjects and patients.

**Table 1:** angle measurements and trans-valvular velocity mapping.

	Volunteers N = 25		Patients N = 25	
Angle measurements	At annulus	At peak velocity	At annulus	At peak velocity

Angle annulus - long axis (angle 1) (degree)	95 (3)	95 (3)	98 (9)	98 (9)

Angle of inflow at E (respectively angle 2 and 3) (degree)	83 (9)	85 (8)	88 (8)	79 (12) ** +

Angle between long axis and inflow at E (difference between angle 1 and 2/3) (degree)	12 (9)	10 (8)	10 (10)	19 (11) ** ++

Angle of inflow at A peak (degree)	77 (8)		85 (12) ++	

Distance annulus level to peak velocity level (mm)		14 (6)		15 (6)

Reformat plane	At annulus	At peak velocity level and angulated perpendicular to inflow	At annulus	At peak velocity level and angulated perpendicular to inflow

Flow volume (ml)	78 (21)	77 (20)	66 (18)+	70 (20) **

Aorta flow (ml)	75 (22)		69 (21)	

Difference with aorta flow (mL and Limits of agreement)	-2.2 (6.1) -14.1;9.6	-2.0 (6.1) -14.1;10.0	2.6 (7.6) -12.3;17.5	-0.9 (4.5) -9.7;7.9

Absolute error (mL)	5.0 (4.5)	4.4 (5.0)	5.9 (5.4)	3.6 (2.7) *

Correlation with aorta flow (R square)	.923	.922	.872	.954

Peak flow rate E (ml/s)	452 (123)	500 (118) **	406 (82)	414 (82) +

Peak flow rate A (ml/s)	192 (67)	194 (72)	201 (66)	201 (70)

Peak velocity E (cm/s)	71 (15)	85 (14) **	68 (19)	94 (25) **

Peak velocity A (cm/s)	36 (18)	41 (18) **	40 (18)	47 (20) *

E/A ratio from peak flow rate	2.5 (0.7)	2.8 (0.8) *	2.2 (1.0)	2.3 (1.1)

E/A ratio from peak velocity	1.8 (0.9)	1.9 (0.9)	1.6 (0.5)	1.8 (0.6) **

Mean (standard deviation) are given with *(p < 0.05) and ** (p < 0.01) indicating statistical significance between measurements at annulus level and at peak velocity level; + (p < 0.05) and ++ (p < 0.01) indicating statistical significant differences between patients and volunteers.

## Conclusions

Streamline visualization of 4DFlow MRI data revealed more laterally orientated LV inflow after AVSD correction. Using this visualization for optimized positioning of the measurement plane (at peak velocity level and perpendicular to inflow direction), assessment of trans-LAVV blood flow proved to be more reliable and resulted in related changes in blood flow characteristics.

## Funding

Willem Alexander Kinder Fonds, Leiden, The Netherlands and Dutch Technology Foundation (STW) project number 11626.

